# Evaluation of the durability of long‐lasting insecticidal nets in Guatemala

**DOI:** 10.1186/s12936-021-03722-1

**Published:** 2021-05-14

**Authors:** María Eugenia Castellanos, Soledad Rodas, José Guillermo Juárez, Juan Carlos Lol, Sayra Chanquin, Zoraida Morales, Lucrecia Vizcaino, Stephen C. Smith, Jodi Vanden Eng, Henok G. Woldu, Audrey Lenhart, Norma Padilla

**Affiliations:** 1 Centro de Estudios en Salud, Universidad del Valle de Guatemala, Guatemala City, Guatemala; 2 Public Health and Tropical Medicine, College of Public Health, Medical and Veterinary Sciences, James Cook University, Townsville, Queensland Australia; 3 Sección de Entomología Médica, Programa de Enfermedades Transmitidas por Vectores, Ministerio de Salud Pública y Asistencia Social, Guatemala City, Guatemala; 4 Sub-programa de Malaria, Programa de Enfermedades Transmitidas por Vectores, Ministerio de Salud Pública y Asistencia Social, Guatemala City, Guatemala; 5 Division of Parasitic Diseases and Malaria, Centers for Disease Control and Prevention, Atlanta, USA; 6 The Center For Health Analytics For National and Global Equity (C.H.A.N.G.E.), Columbia, USA

**Keywords:** Insecticide-treated bed nets, Malaria, Long-lasting insecticidal nets, Durability, Deltamethrin, attrition, Survivorship, Bio-efficacy, Insecticide content

## Abstract

**Background:**

Insecticide-treated bed nets (ITNs) are widely used for the prevention and control of malaria. In Guatemala, since 2006, ITNs have been distributed free of charge in the highest risk malaria-endemic areas and constitute one of the primary vector control measures in the country. Despite relying on ITNs for almost 15 years, there is a lack of data to inform the timely replacement of ITNs whose effectiveness becomes diminished by routine use.

**Methods:**

The survivorship, physical integrity, insecticide content and bio-efficacy of ITNs were assessed through cross-sectional surveys conducted at 18, 24 and 32 months after a 2012 distribution of PermaNet® 2.0 in a malaria focus in Guatemala. A working definition of ‘LLIN providing adequate protection’ was developed based on the combination of the previous parameters and usage of the net. A total of 988 ITNs were analysed (290 at 18 months, 349 at 24 months and 349 at 32 months).

**Results:**

The functional survivorship of bed nets decreased over time, from 92% at 18 months, to 81% at 24 months and 69% at 32 months. Independent of the time of the survey, less than 80% of the bed nets that were still present in the household were reported to have been used the night before. The proportion of bed nets categorized as “in good condition” per World Health Organization (WHO) guidelines of the total hole surface area, diminished from 77% to 18 months to 58% at 32 months. The portion of ITNs with deltamethrin concentration less than 10 mg/m^2^ increased over time. Among the bed nets for which bioassays were conducted, the percentage that met WHO criteria for efficacy dropped from 90% to 18 months to 52% at 32 months. The proportion of long-lasting insecticidal nets (LLINs) providing adequate protection was 38% at 24 months and 21% at 32 months.

**Conclusions:**

At 32 months, only one in five of the LLINs distributed in the campaign provided adequate protection in terms of survivorship, physical integrity, bio-efficacy and usage. Efforts to encourage the community to retain, use, and properly care for the LLINs may improve their impact. Durability assessments should be included in future campaigns.

**Supplementary Information:**

The online version contains supplementary material available at 10.1186/s12936-021-03722-1.

## Background

Globally, insecticide-treated bed nets (ITNs) are a standard intervention for the prevention and control of malaria. Thirteen of the seventeen malaria-endemic countries in the Americas have incorporated within their malaria control and prevention programmes the universal distribution of ITNs, and report having a policy of distributing ITNs through mass campaigns [1].

The use of ITNs has had a significant impact on personal protection and in reducing malaria transmission. If high ITN coverage is sustained (> 80%), nets can provide protection to the larger community, including those households that do not use them [2]. The Global Malaria Programme of the World Health Organization (WHO) recommends universal access and use of ITNs as a primary intervention for malaria control [3].

Previous assessments of ITNs show a high degree of variation in ITN durability [4–6], with ITNs from the same manufacturer showing significantly different levels of durability. The factors that influence this variability include the frequency of washing (including the quality of the water), type of laundry soap, washing and drying techniques, and daily levels of “wear and tear”. Translating this variability into a single 3–5 year durability estimate for ITNs could result in an inaccurate presumption of ITN effectiveness in areas where durability is compromised considerably faster. As such, understanding durability is a key consideration for the timing of ITN replacement strategies, and should be a component of any distribution campaign.

While ITN durability has been much more broadly studied in Africa, limited data are available from the Americas. An ITN distribution took place in Guatemala in 2012–2013 as part of malaria control activities supported by the Global Fund to Fight AIDS, TB and Malaria. Approximately 929,000 PermaNet® 2.0 long-lasting insecticidal nets (LLINs) were distributed in 14 Health Areas that comprise a total of 111 municipalities in 12 departments of Guatemala. The primary objective of this study was to assess the durability of the PermaNet® 2.0 LLINs over 32 months of routine use in an area with high malaria burden in the Mesoamerican region. The three elements of durability, survivorship, physical integrity and insecticidal activity were evaluated, following the WHO guidelines for monitoring the durability of ITNs under operational conditions [7]. This evaluation was designed to provide relevant data for future plans around ITN replacement strategies that could be relevant in both Guatemala and more broadly in Latin America.

## Methods

### Study site and description of the ITN distribution

**Fig. 1:**
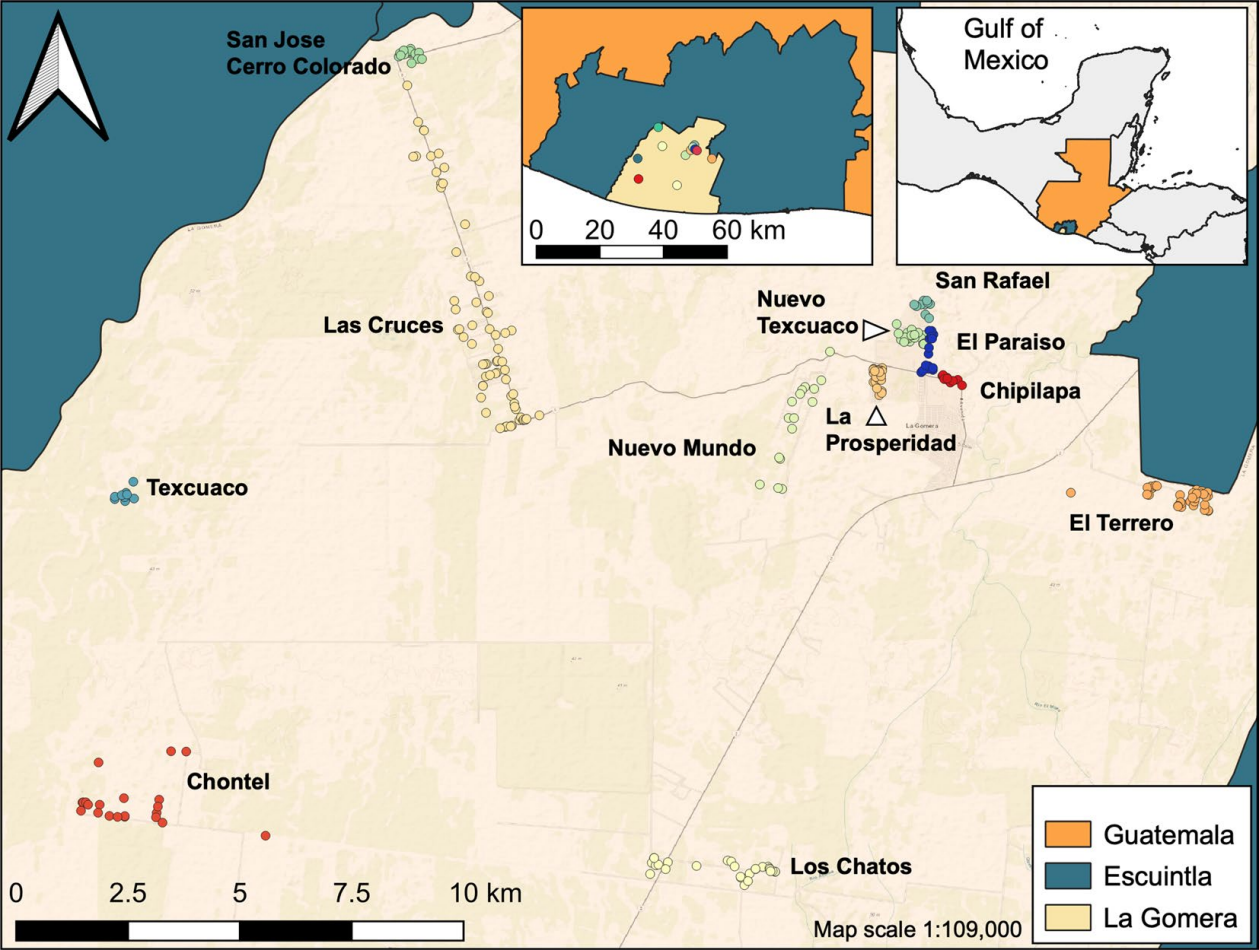
Location of the 12 study communities in La Gomera, Escuintla, Guatemala

The study was conducted in the municipality of La Gomera in the department of Escuintla in southern Guatemala (Fig. 1). Escuintla was included in the 2012–2013 ITN mass distribution campaign. According to national surveillance data, 56% of all malaria cases in Guatemala are reported from Escuintla (2012). As of 2012, La Gomera is divided into 19 communities, had a population of 60,299 inhabitants and reported 23% of the malaria cases in the country (PAHO, 2017). In Escuintla, malaria is mainly transmitted by *Anopheles albimanus* and *Plasmodium vivax* is responsible for over 95% of the cases. 69,214 PermaNet® 2.0 (Vestergaard Frandsen, Lausanne, Switzerland) deltamethrin-treated LLINs were distributed in Escuintla from April 2012 to September 2013. It was the first mass LLIN distribution campaign in the region.

### Experimental design

The evaluation was carried out from November 2013 to March 2015 through three prospective cross-sectional surveys in randomly selected households using a multistage sample design. The first stage of the sampling consisted of a non-probabilistic sampling during which one municipality within the LLINs distribution area was selected (La Gomera). The following stage was done using a cluster survey methodology with each cluster size proportional to its population. The evaluation included only those communities that received LLINs approximately 18 months (range = 17–19 months) prior to the first survey. Twelve communities met this criterion and were included in the 18-and 24-months survey. In these twelve communities, 4,076 LLINs had been delivered in the study area between April-June 2012. At the 32-month survey, only 8 of these 12 communities were included since in the other four communities a round of LLIN replacement occurred in 2014. Nets were not labelled prior to distribution and, therefore, users were not aware that the nets were to be evaluated, reducing the possibility of the Hawthorne effect [8].

### Sample size, selection of households and nets evaluated

The study population was defined as the population that received nets based on the Ministry of Health (MoH) 2012 LLINs campaign distribution list (MoH list). This list was generated during the distribution campaign with information of family name, sleeping spaces, number of nets delivered to the household. The household then received a number of nets equal to the number of reported sleeping spaces. The MoH distribution campaign was intended to have a 100% coverage of the sleeping spaces in the localities selected for distribution.

To determine survivorship and physical integrity of the LLINs, sample size estimates were based on the confidence interval around the attrition rate at each time period, according to values reported for Kenya (11% for 18 months, 15% for 24 months, 23% for 30 months) [9]. A relative error of 5%, a confidence level of 95%, and a design effect of 2.0 were assumed. Initially a non-response proportion of 15% at 18 months, 20% for the 24 months and 25% for 36 months was assumed. However, for the surveys conducted at 24 months and 32 months this proportion was increased to 40 and 55%, respectively, as a different approach was used to identify the households.

#### 18-month household selection

 Households were identified using the MoH list. The OpenEpi Random Program (www.openepi.com) was used to generate a random number list for the final selection of households. The addresses of the households provided by the MoH list were used to locate the household. However, rural communities in Guatemala, like the study site, did not use conventional addresses and it was found that the list contained incomplete addresses making extremely challenging to locate the randomly selected households. A decision was made to change the method for household selection for the 24-month and 32-month surveys.

#### 24-month and 32-month household selection

 Aerial Google Earth imagery and the MoH list were used to identify houses in each community for sampling (Map data: Google, DigitalGlobe). The high resolution of the 2013 images of the selected communities allowed for good accuracy when mapping individual houses. The Google Earth file was imported to ArcMap 9.3 (ESRI, Redlands, CA, USA) using a KML to shapefile extension [10]. Longitude and latitude coordinates (WGS84 coordinate systems) were added to the attributes table which was saved as a dBASE file. This was imported to an Excel spreadsheet for the random household selection, using simple random sampling with the OpenEpi Random Program, using the initial non-response rates. After the localization of a household, it was verified that the household had received campaign LLINs based on the MoH campaign distribution list and if so, the number of nets received. It was detected at that stage that a high proportion of households were not part of the list, with the household members confirming they did not receive them, resulting in a re-adjustment of the non-response rate leading to an increase the number of households randomly selected to locate. Also, for the surveys at 24 months and 32 months, there was an additional increase of 5% of the number of households to survey because the use of Google Maps might result in some structures that had been enumerated as households and were another type of structure [11].

After these re-adjustments, the numbers of households selected to survey at each time point were as follows: n = 348 at 18 months, n = 583 at 24 months, and n = 887 at 32 months, for a total of 1,818 households.

### Questionnaires

Programme staff from the Vector-borne Diseases Programme of Escuintla and researchers from Universidad del Valle de Guatemala conducted the surveys. The survey teams were trained to understand the structure of the survey and how to deliver it using the personal digital assistants (PDAs) that included a GPS element. For the 24 and 32-month surveys, the geographic information system (GIS) information about each selected community was uploaded to GPS Visualizer [12](www.gpsvisualizer.com), and subsequently added to a Garmin global positioning system (GPS) map 62 S (Garmin, USA) with a 20 m proximity alarm. The interviewers used this instrument to locate the households.

Survey questions were based on WHO Durability guidelines [7], adapted to local settings, translated to Spanish and validated. The questionnaire was administered primarily to the adult woman in charge of the household in order to collect as much data as possible on the maintenance and washing frequencies of the LLINs. If an adult woman was not present, then the questionnaire was administered to any adult household member. Questions were included on (a) demographic characteristics of the enrolled household; (b) net survivorship, attrition and use; and (c) physical integrity of the fabric measured *in situ*. Of the total number of LLINs that were delivered to the household, one was randomly selected for further analysis (1 enrolled household = 1 enrolled LLIN). At the end of the questionnaire, the enrolled net was double labeled, with an indelible fabric marker and with a plastic loop seal. A different colour was used to identify nets enrolled in each of the surveys (time-points). Questions on net use and physical integrity were directed towards this randomly selected specific net if it was still present in the household. If the selected LLIN was no longer present, other questions related to reasons for its absence were pursued. In the 24-month and 32-month surveys, if a previously surveyed household was selected, a LLIN was still randomly selected. If the selected LLIN had already been evaluated, another one was selected if available.

### Physical integrity

LLINs were assessed *in situ* with the net left hanging over the sleeping space if the position allowed it. In the cases that the LLIN position did not allow an accurate hole counting, the survey team asked the LLINs owners to take down the net and extend it so the interviewers can count the holes with precision. To facilitate the hole counting in each net panel and to reduce the number of errors that can derive from misunderstandings of the panel to count, the survey team followed a strict order that was shown in the PDA: roof, head panel, lateral head panel-right, feet panel, lateral head panel-left. Holes were classified according to the WHO 2011 categories: (1) holes smaller than a thumb (~ 0.5 to 2 cm diameter), (2) bigger than a thumb but smaller than a fist (~ 2 to 10 cm diameter), (3) bigger than a fist but smaller than a head (~ 10 to 25 cm diameter) and (4) bigger than a head ( > ~ 25 cm diameter) [7]. A standard ruler was available in case there was uncertainty on a measurement.

### Insecticide content

Deltamethrin content, in mg/m^2^, was measured using a Tracer III-SD handheld X-ray fluorescence (XRF) analyser (Bruker Nano Analytics, Inc., Kennewick, WA, USA). For this analysis, deltamethrin content was calculated from the intensity of 11.549–12.248 keV X-rays emitted by the bromine atoms of the deltamethrin in the sample [13]. Calibration samples of polyester mosquito netting treated with six different levels of deltamethrin (0–114 mg/m^2^) were used to correlate intensity with deltamethrin content. The correlation was linear with R^2^ = 0.98, minimum. For whole bed net analysis, the net was folded in a way to create a 24-layer sampling point, comprising 8 points from the roof of the net, and 4 points from each of the 4 sides. This multilayer sample was then analysed to yield the average deltamethrin content over the 24 locations. Since folding the net and collecting the data takes only 2–3 min, the average deltamethrin content of many nets can be measured quickly and conveniently [14, 15].

### Sample size for cone bioassays

From the LLINs evaluated in the households in each community, a sub-sample of the nets ´still in the household and which can be used for sleeping under´was randomly selected and collected for bio-efficacy testing. In order to detect the loss of at least 5.6% of insecticide, with respect to the baseline level of 55 mg/m^2^, a power of 90%, alpha = 0.05, and using a standard deviation of 5, a subset of 55 LLINs were randomly selected during the 18-month survey, 60 at 24 months, and 65 at 32 months, with increasing number of nets selected in order to compensate for an estimated non-response effect of 15%.

Collected nets were placed individually in plastic bags and grouped inside large black polyethylene bags and kept approximately at 21 °C until their arrival to the laboratory. A replacement net from the same brand and size was given to the household. The net was labeled to differentiate it from the nets distributed during the campaign.

### WHO cone bioassays

Cone bioassays were performed on samples from each of the five sides of each bed net and following the sampling scheme recommended by the WHO [7]. Until processed the net samples were stored at 4 °C wrapped in aluminum foil and placed inside plastic bags. Briefly, 2–3-day old, non-blood fed *Anopheles albimanus* females from the laboratory insecticide susceptible SANARATE strain were used. Two replicates of 20 mosquitoes were exposed to each of the five PermaNet® 2 net pieces corresponding to the roof and each of the 4 sides panels as established by the WHO in 2011 and two replicates of 10 mosquitoes were exposed to an untreated polyester net as control. Mosquitoes were exposed for 3 min and the 60 min knock-down and 24-h mortality recorded according to WHO criteria. Data were adjusted according to Abbot's formula. Bioassays were performed under laboratory conditions, 27 ± 2 °C and 70 ± 10% relative humidity. Knock down at 60 min (KD60) and mortality at 24 h were calculated as the number of mosquitoes knocked down at 60 min and the number of dead mosquitoes at 24 h proportional to the total number exposed. Results were considered valid if the control mortality was lower than 5%; between 5 and 20% control mortality, results were corrected using Abbott´s formula and if control mortality was greater than 20%, the bioassays for that net were repeated [7].

### Verification of baseline LLIN insecticidal content

From the PermaNet® 2.0 batch distributed in 2012, 11 nets were retained and analysed using XRF and WHO cone bioassays at 24 months post-distribution to assess the insecticidal content of the unused LLINs (considered time-point 0). For a period of 20 months, the mosquito nets were stored in their original packaging in an unregulated warehouse in Escuintla (without humidity and temperature control). In November 2013, nets were transferred to the UVG where they were stored in an environment at 21 °C until processed.

### Data analysis

The main outcomes of the study were overall and functional survivorship, physical integrity, insecticide content measured by XRF and bio-efficacy measured by the WHO cone bioassay. The original data analysis plan intended to follow the WHO 2011 guidelines for monitoring the durability of ITNs under operational conditions [7]. The data analysis plan was updated after the publication of new technical documents and research articles [16–18].

Proportions and measures of central tendency were used to estimate descriptive demographic characteristics of the households that were enrolled and details of their reported use and handling of the LLINs at the three-survey time-points (18 months, 24 months and 32 months).

The overall survivorship at each time-point was defined as the number of enrolled LLINs still present in the households divided by the total number of enrolled LLINs. The functional survivorship was calculated at each time-point as follows: number of enrolled LLINs still present and “serviceable” (see definition below) in the households divided by the number of LLINs still present + LLINs not present owed to attrition reasons (damaged and thrown away or LLIN used for other purposes) [17, 19]. The median survival time, defined as “the time point at which the estimate of functional LLIN survival crosses the 50% mark”, [17] was calculated using the following formula: $tm={t}_{1}+\frac{\left({t}_{2}-{t}_{1}\right)\times ({p}_{1}-50)}{\left({p}_{1}-{p}_{2}\right)}$, where *tm* is the median survival time, t_1_ and t_2_ were the 24 and 32 month survey points (in years) and p_1_ and p_2_ were the functional survivorship proportions for the 24 and 32 month points, respectively.

The physical integrity of LLINs was measured by estimating the proportion of LLINs with at least one hole of any size, the total number of holes/LLIN, and the total hole area (cm^2^)/ LLIN in quartiles. The total hole surface area (THSA) was calculated by multiplying the number of holes by the area of each hole (1.2, 28.3, 240.5 or 706.9 depending on the size category of the holes) and then summing across the categories [16, 18]. Holes smaller than 0.5 cm were not included in the assessment. The LLIN condition was classified as good (< 79 cm^2^ THSA), damaged (79–788 cm^2^ THSA) or severely torn ( > = 789 cm^2^ THSA) following the categorization recommended by WHO, and an LLIN was considered “serviceable” if it was classified as good or damaged [17]. The proportion of LLINs with evidence of repairs was also calculated.

The median concentration and 95% CI of deltamethrin (mg/m^2^) obtained through the XRF analyses was estimated at each time point, including unused nets. The proportions of LLINs with a concentration of less than 10 mg/m^2^ and 25 mg/m^2^ were calculated. These values were chosen because LLINs with less than 10 mg/m^2^ are considered to not have the minimum effective concentration of insecticide, and nets with less than 25 mg/m^2^ are considered to not contain an optimum insecticide concentration [14].

For the bio-efficacy results, the distribution and geometric mean (CI 95) for the KD60 and mortality at 24 h were estimated, for each survey time point including unused nets [19]. The proportions (and CI 95) of LLINs that showed ≥ 80% mortality at 24 h, or ≥ 95% KD60 at each time point, including for unused nets, were also calculated [18].

The association between the cone bioassay results and the deltamethrin concentration obtained by the XRF was assessed using Locally Weighted Scatterplot Smoothing (LOESS) regression analysis to estimate Kendall’s correlation coefficient. A predictive model was developed using piecewise (segmented) regression in which it was explored if the use of the deltamethrin concentration measured by XRF and adjusted by other variables (More details in Additional file 1) can predict if results of a bioassay on the LLIN will exceed or not the WHO threshold of ≥ 80% mortality at 24 h.

Nets were analysed based on a series of sequential criteria: (a) Distributed in the household, (b) Still present in the household, (c) Used at least once, (d) Used the night before the survey, (e) In serviceable condition and (f) With a concentration of deltamethrin above 10 mg/m^2^ as measured by XRF (this criterion was not included the 18-month survey as only a small number of LLINs were analysed with XRF). For the 24- and 32-month surveys, a working definition of ‘LLIN providing adequate protection’, for nets that met all of these criteria was developed. For this purpose, the proportion of LLINs that sequentially meet each criterion was estimated. In order to be analysed in each criterion, the LLINs should have met the requirement of the previous criterion. Then the overall proportion of LLINs providing adequate protection was estimated based on the study definition.

The Cochran-Armitage Trend Test was used to assess trends across the time-points and the different categorical outcomes. For continuous outcomes, the Kruskal-Wallis test (differences among all groups) was used. The difference in medians among specific groups was compared by estimating its 95% CI intervals by bootstrapping, setting the number of bootstraps replicates to 10,000, using the ‘boot’ package [20]. All analyses were conducted using SAS software v 9.4 (SAS Institute, Cary, NC, US) and R v3.6.0 (R Foundation for Statistical Computing, Vienna, Austria, 2016).

## Results

### Descriptive characteristics of the study population

**Fig. 2:**
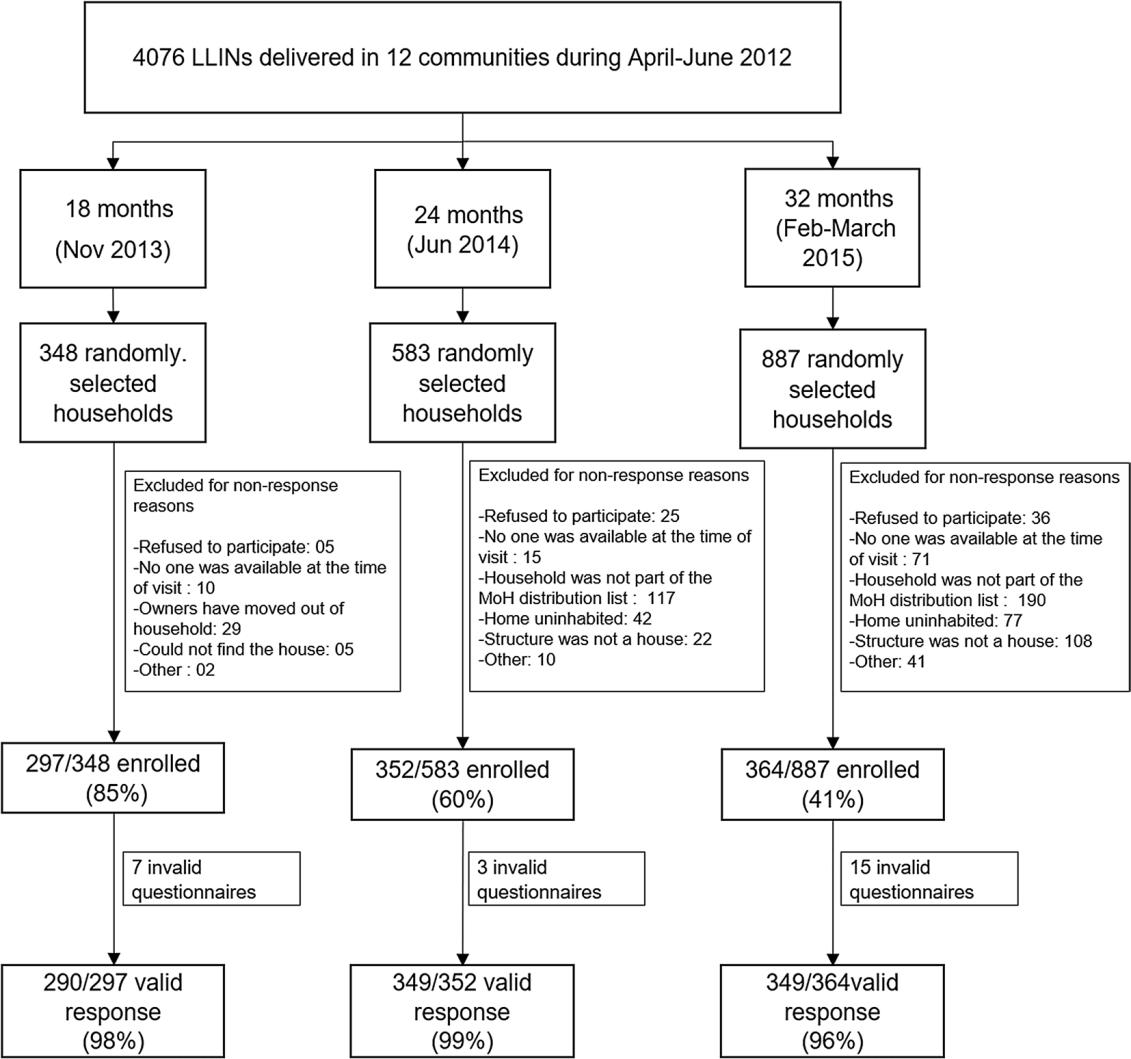
Flow chart of the data collection and number of long-lasting insecticidal nets (LLINs) that were evaluated for each of the measurements. Invalid questionnaires represent questionnaires with incomplete or discrepant data or questionnaires carried out on nets that were not part of the PermaNet® 2.0 2012 distribution

A total of 1,013 LLINs were enrolled in the study, of which 988 (98%) of them were considered to have valid information. Of these, 290 were evaluated at 18 months, 349 at 24 months and 349 at 32 months (Fig. 2). The education level of the head of household and the proportion of households with electricity that participated in the surveys remained consistent throughout the duration of the study (Table 1). A two-fold increment was observed in the proportion of households that owned a flush toilet and that had piped water as their source of drinking water in the 32-month survey as compared with the previous time points. The median number of individuals that slept in the household the night before the survey was four (Additional file 1: Table S1). According to the census of LLINs distributed, each household received a median of three LLINs, which corresponded to the median number of sleeping places used in the household the night before of the survey.

**Table 1:** Demographic characteristics of the surveyed households and head of household at the three time-points

Characteristic	18 months n = 290	24 months n = 349	32 months n = 349
Education level head of household			
None (n, %)	84 (29)	79 (23)	99 (28)
Primary school through 3rd grade (n, %)	65 (22)	93 (27)	102 (29)
Primary school through 6th grade (n, %)	88 (30)	99 (28)	92 (26)
Basic level (n, %)^a^	24 (8)	36 (10)	29 (8)
Diversified level (n, %)^b^	16 (6)	30 (9)	20 (6)
Higher education/University (n, %)	4 (1)	1 (0)	6 (2)
Does not know/unclear (n, %)	9 (3)	11(3)	1 (0)
Mother as the respondent (n, %)	216 (74)	269 (77)	270 (77)
Electricity present (n, %)	265 (91)	338 (97)	343 (98)
Own flush toilet (n, %)	75 (26)	99 (28)	187 (54)
Piped water into home (n, %)	77 (27)	77 (22)	147 (42)

^a^ Secondary education, first three years

^b^ Secondary education, last two or three years

### Overall and functional survivorship

**Fig. 3:**
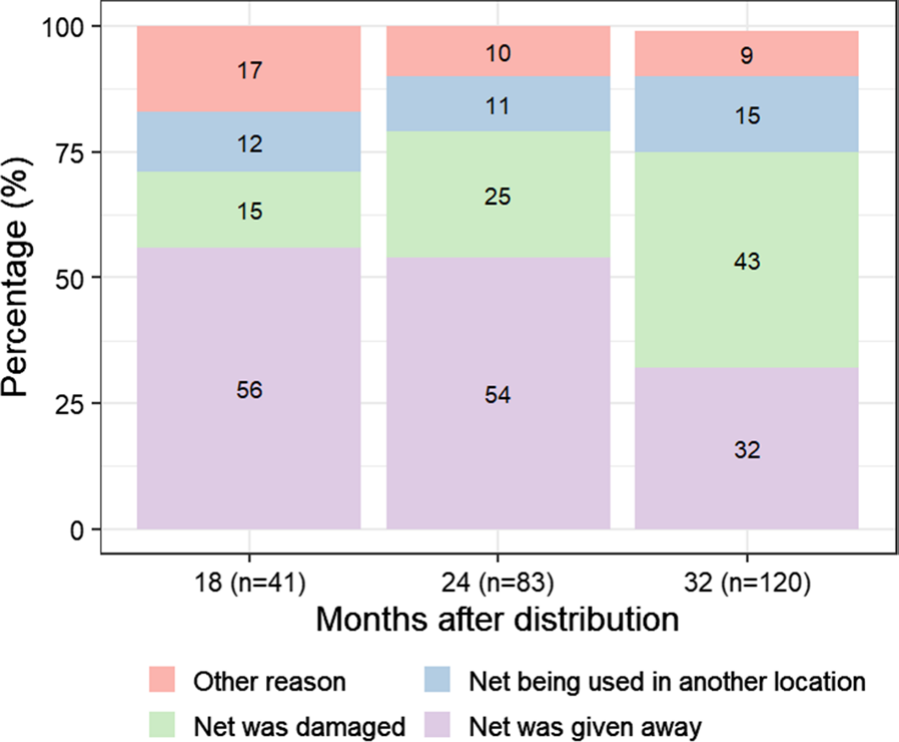
Reasons for long-lasting insecticidal nets (LLINs) loss

The proportion of LLINs present in the household decreased over time (p < 0.001), with an overall survivorship of 86% (CI 95: 82–90) at 18 months, 76% (CI 95: 72–81) at 24 months and 66% (CI 95: 61–71) at 32 months. The main reported cause of loss at 18 months (56%, CI 95: 40–72) and 24 months (54%, CI 95: 43–65) was that the net was given away to others, whereas at the 32-month survey, the main cause was that the net was damaged and thrown away (43% CI 95: 34–52) (Fig. 3 and Additional file 1: Table S2). The functional survivorship was 92% (CI 95: 88–95) at 18 months and 81% (CI 95: 76–86) at 24 months. The median survival time 3.7 years (CI 95: 3.4–4.2), with 69% (CI 95: 64–75) still functionally present at 32 months.

### LLIN use and handling

More than 90% of the LLINs still present in the households were reported to have been used at least once at any of the time points (93% at 18 months, 97% at 24 months and 95% at 32 months). However, among the LLINs used at least once, less than 80% were reported to have been used the night before the survey (74% at 18 months, 79% at 24 months and 71% at 32 months) (Table 2). Almost a third of these LLINs were found not hanging (stored away or visible but not hung up) at each of the survey time-points.

**Table 2:** Long-lasting insecticidal nets (LLINs) use and handling as reported by householders at each survey time-point

	18 months n = 249	24 months n = 266	32 months n = 229
LLIN used at least once to sleep (n, %)^a^	231 (93)	257 (96)	217 (95)
LLIN used the night before the survey (n, %)	171 (74)	203 (79)	154 (71)
LLIN used every night during the week before the survey (n, %)	189 (82)	208 (81)	167 (77)
LLIN used year-round (n, %)	205 (89)	215 (84)	178 (82)
LLIN tucked under bed at night (n, %)	213 (92)	241 (94)	194 (89)
LLIN observed at time of survey, among LLIN used at least once to sleep			
Hanging loose over sleeping place (n, %)	66 (29)	87 (34)	69 (32)
Hanging tied in knot (n, %)	17 (7)	33 (13)	35 (16)
Hanging folded (n, %)	37 (16)	28 (11)	24 (11)
Visible but not hanging (n, %)	13 (6)	15 (6)	10 (5)
Stored away (n, %)	53 (23)	56 (22)	60 (28)
Hanging next to the wall (n, %)	45 (19)	38 (15)	19 (9)
Washing activities, among LLIN used at least once to sleep			
Ever washed ^b^ (n, %)	204 (88)	237 (92)	209 (96)
Bed net washed < 1 month ago (n, %)	128 (63)	131 (55)	121(58)
When washed, soaked (n, %)	130 (64)	147 (62)	126 (60)
Detergent used (n, %)	148 (72)	189 (80)	163 (78)
When washed, scrubbed hard (n, %)	127(62)	122 (51)	97(46)
Dried outside in the sun (n, %)	143 (70)	163 (69)	126 (60)
Dried outside in the shade (n, %)	54 (26)	70 (30)	75 (36)
Dried inside (n, %)	5 (2)	3 (2)	4 (2)

*LLIN * long-lasting insecticidal nets, *IQR *Interquartile boundary values (p25, p75)

^a^This ‘n’ serves as the denominator for the subsequent categories of use and position of LLIN observed at time of survey

^b^ This ‘n’ serves as the denominator for the subsequent washing categories

A large proportion of the LLINs that were used at least once had been washed (88% at 18 months, 92% at 24 months, 96% at 32 months) and in > 55% of the cases, nets had been washed less than 1 month preceding the survey (Table 2). Among the washed LLINs, the majority were soaked (64% at 18 months, 62% at 24 months, 60% at 32 months), although in over 70% of these, the reported soaking time was less than one hour (80% at 18 months, 73% at 24 months, 74% at 32 months). The principal soap used to wash the LLINs was detergent powder, either alone or in combination with bar soap and/or bleach. Over time, there was a reduction in the proportion of LLINs that were scrubbed hard or beaten on a hard surface during washing (62% at 18 months, 51% at 24 months and 46% at 32 months, p = 0.0007). In most cases, the washed LLINs were dried outside in the sun (70% at 18 months, 69% at 24 months and 60% at 32 months).

### Physical integrity

The median number of holes in the LLINs was 2 (IQR 0,7) at 18 months, 4 (0,15) at 24 months and 5.5 (IQR 1,16) at 32 months (Table 3). The median total hole area (cm^2^) in the bed nets was 2.4 (IQR 0,63.8) at 18 months, 8.4 (IQR 0,271.5) at 24 months and 34.3 (IQR 1.2,327.8) at 32 months. At 32 months, 84% of the LLINs were serviceable and 58% were in good condition.

**Table 3:** Physical integrity of long-lasting insecticidal nets (LLINs), used at least once for sleeping and with complete data regarding holes

Characteristic	18 months n = 222	24 months n = 256	32 months n = 214
Any holes (n, %)	131 (59)	181 (71)	162 (76)
Any holes repaired (n, %)^a^	30 (23)	42 (23)	71 (44)
By stitches (n, %)	23 (77)	27 (64)	54 (76)
By knots (n, %)	9 (30)	20 (48)	33 (46)
By patches (n, %)	1 (3)	0 (0)	1 (1)
Median total number of holes [IQR]	2 [0, 7]	4 [0,15]	5.5 [1, 16]
Median total hole area (cm^2^) [IQR]	2.4 [0, 63.8]	8.4 [0, 271.5]	34.3 [1.2, 327.8]
Categorization based on total hole area (cm^2^)			
Serviceable condition^b^ *(n, %)*	208 (94)	225 (88)	180 (84)
Good condition (n, %)	171 (77)	180 (70)	125 (58)
Damaged condition (n, %)	37 (17)	45 (18)	55 (26)
Too torn condition (n, %)	14 (6)	31 (12)	34 (16)

*IQR* Interquartile boundary values (p25, p75)

^a^ This ‘n’ serves as the denominator for the type of repair categories

^b^Serviceable condition includes long-lasting insecticidal nets with good or damaged condition

Respondents reported new holes in the month prior to the survey in 24% of bed nets at 18 months, in 15% of bed nets at 24 months and in 27% at 32 months. The principal reported cause of these new holes was that the bed net tore or was split when caught on an object (45% at 18 months, 31% at 24 months and 38% at 32 months). Other reported causes of holes were children (21% at 18 months, 10% at 24 months and 19% at 32 months) and animals (11% at 18 months, 13% at 24 months and 10% at 32 months). However, a large proportion of these new holes did not have a known cause reported by the respondent (21% at 18 months, 36% at 24 months and 24% at 32 months). No new holes were reported due to burns. A minority of the LLINs with holes had been repaired (23% at 18 and 24 months, 44% at 32 months), with stitches as the principal mode of repair (Table 3).

### Insecticide content

**Fig. 4:**
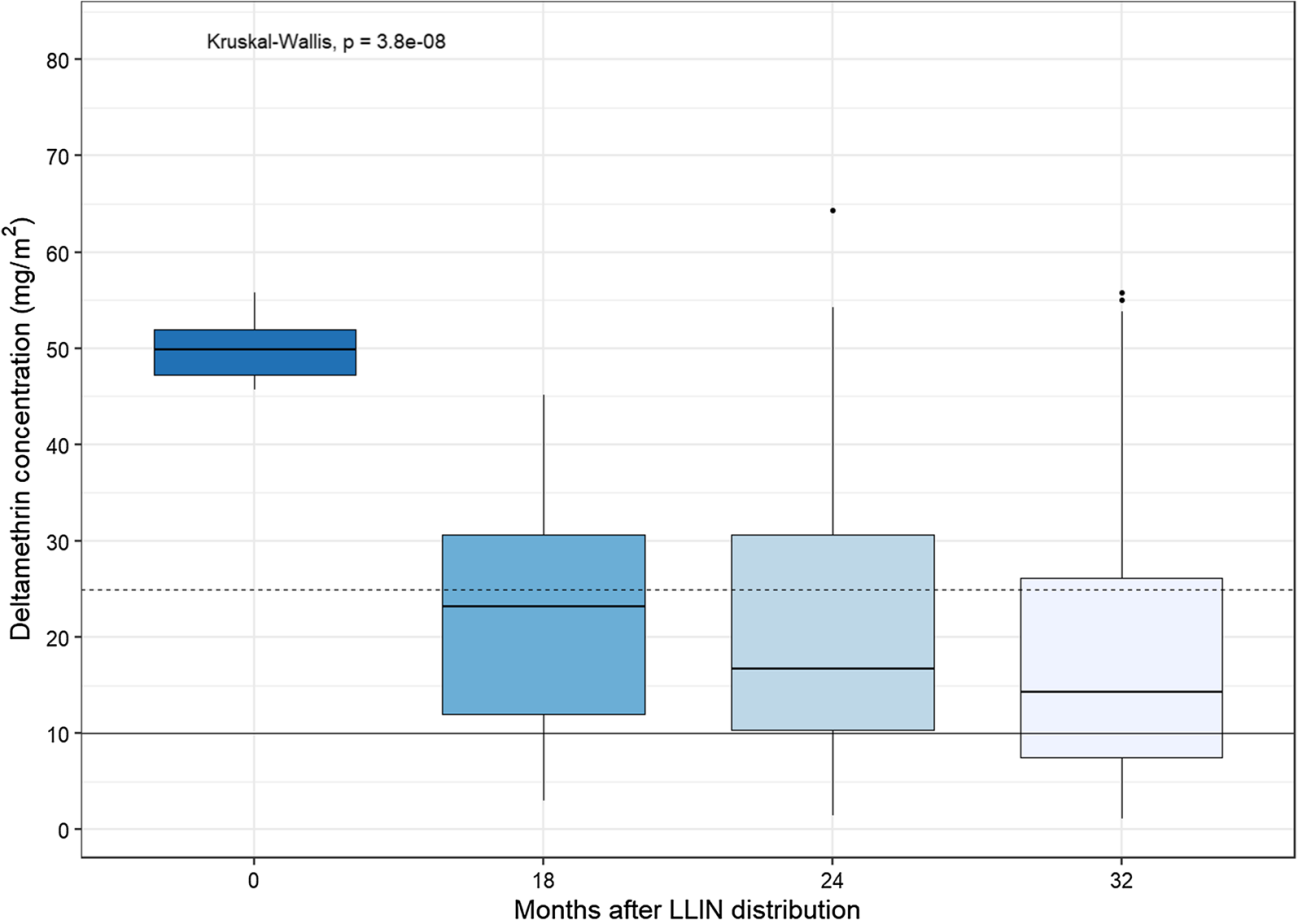
Estimated total deltamethrin concentration (mg/m^2^) measured by X-ray fluorescence (XRF) at the survey time-points. The time-point of 0 represents values on unused long-lasting insecticidal nets (LLINs) that were from the same batch as those distributed. The dashed horizontal line represents the threshold of 25 mg/m^2^ and the solid horizontal line represents the threshold of 10 mg/m^2^

The median deltamethrin concentrations (mg/m^2^) measured by XRF differed significantly by survey time-point (p < 0.0001) (Fig. 4). For unused nets, the median was 49.8 mg/m^2^ (CI 95 46.2–55.3, n = 11), at 18 months it was 23.2 mg/m^2^ (CI 95 14.6–26.6, n = 51), at 24 months it was 16.7 mg/m^2^ (CI 95 14.9–18.3, n = 264) and at 32 months it was 14.4 mg/m^2^ (95 CI 12.8–17.4, n = 218). The reductions in insecticide content were statistically significant between unused nets and values measured at 18 months (median difference: 26.4 mg/m^2^, CI 95 22.1,35.3) but not between 18 months to 24 months (median difference: 6.3 mg/m^2^, CI 95 − 1.5, 10.8) neither between 24 months and 32 months (median difference: 2.2 mg/m^2^, CI 95 − 0.85, 4.95). The proportion of LLINs with a deltamethrin concentration of less than 10 mg/m^2^ increased over time: 0% for unused nets, 14% (CI 95: 4–24) at 18 months, 23% (CI 95: 18–29) at 24 months and 35% (CI 95: 29–42) at 32 months (p < 0.0001). When the cut-off was set at 25 mg/m^2^, the proportion of LLINs below the threshold was: 0% for unused nets, 57% (CI95: 43–71) at 18 months, 69% (CI 95: 63–75) at 24 months and 73% (CI 95: 67–79) at 32 months (p < 0.0001).

### Bio‐efficacy

For the unused nets (n = 11), the geometric mean KD60 was 100% (95% CI 99–100) and the geometric mean mortality at 24 h was 100% (CI 95 100–100) (Additional file 1: Figures S1 and S2). For the 18-month nets (n = 51), the geometric mean KD60 was 96% (95% CI 94–99) and the geometric mean mortality at 24 h was 82% (CI 95 76–89). For the 24-month nets (n = 60), the geometric mean KD60 was 80% (95% CI 73–87) and the geometric mean mortality at 24 h was 52% (CI 95 43–61). For the 32-month nets (n = 63), the geometric mean KD60 was 80% (95% CI 73–87) and the geometric mean mortality at 24 h was 45% (CI 95 35–55).

On summary, the proportion of LLINs that passed the WHO ≥ 80% mortality threshold or ≥ 95% KD60 for bio-efficacy were 100% for unused nets, 90% (CI 95 82–99) at 18 months, 68% (CI 95 56–80) at 24 months and 52% (CI 95 40–65) at 32 months (p < 0.0001).

### Association between mortality at 24 h and deltamethrin concentrations (mg/m^2^) measured by XRF

**Fig. 5:**
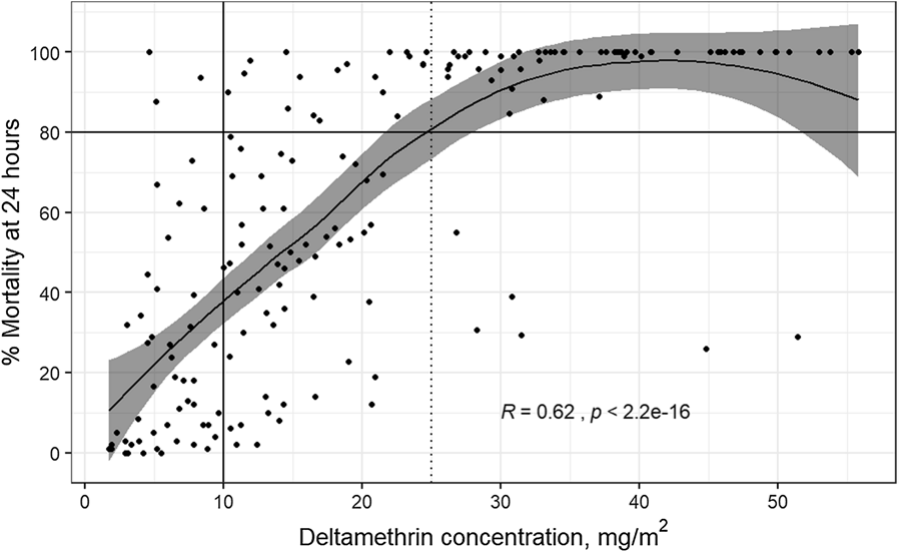
Locally weighted regression (LOESS) analysis between results of the cone bioassays measuring percent mortality at 24 h and concentration of deltamethrin (mg/m^2^) as measured by X-ray fluorescence (XRF) analysis for all survey time-points. Each dot represents one long-lasting insecticidal net (LLIN) plotted based corresponding cone bioasays and XRF measurements. The gray area represents the 95% confidence interval. The solid vertical line represents the threshold of 10 mg/m^2^ and the dashed vertical line represents the threshold of 25 mg/m^2^. The black horizontal line represents the 80% mortality threshold

There was a positive linear relationship between deltamethrin concentration measured by XRF and the percent mortality at 24 h up to approximately 25 mg/m^2^ of deltamethrin concentration (r = 0.62, p < 0.0001) (Fig. 5 and Additional file 1: Fig. S3). The summary statistics from the fitted segmented regression model confirmed the positive relationship between deltamethrin content and mosquito mortality (Additional file 1: Table S3), with an estimated breakpoint at 24.4 mg/m^2^. The age of the LLINs was also an independent predictor of mosquito mortality (%) after 24 h. The mortality decreased with increasing age of the LLIN. This model using segmented regression was able to predict 89.7% of the time if a LLIN will achieve the WHO 80% of mortality threshold for bio-efficacy using only two predictors: insecticide content measurements obtained from bromine x-ray fluorescence testing and the age of the LLINs.

### Overall proportion of LLINs providing adequate protection

**Fig. 6:**
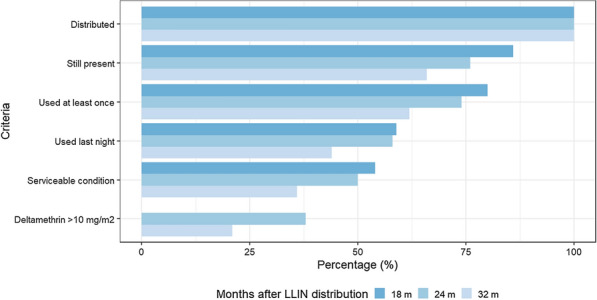
Estimate of effective protection of long-lasting insecticidal nets (LLINs) in La Gomera, Escuintla. The proportion of LLINs that met each of the criteria listed on the left is shown in sequential descending order, with each value shown as a subset of the criteria above. If the LLIN was able to fulfill all criteria, it was considered a LLIN that provided adequate protection. The stage of deltamethrin above 10 mg/m^2^ as measured by XRF for the 18-month survey was not included as only a small number of LLINs were analysed with XRF at that time-point

The proportion of LLINs providing adequate protection was 38% (CI 95 33–44) at 24 months and 21% (CI 95 17–26) at 32 months. Three components contributed most to the observed decreases in adequate protection: (1) A decrease between the number of LLINs distributed and the proportion of LLINs still present in the household-which was particularly important at 32 months (drop of 34%); (2) A reduction between the proportion of LLINs present and used at least once to the proportion of LLINs present and used the night before of the survey (drop of 21%, 16 and 18% for 18, 24 and 32 months, respectively); and (3) A drop between the proportion of LLINs in serviceable condition and the proportion of LLINs in serviceable condition with a deltamethrin concentration of at least 10 mg/m^2^ (drop of 12 and 15% for 24 and 32 months, respectively) (Fig. 6 and Additional file 1: Table S4).

## Discussion

In this prospective multiple cross-sectional study, the durability of PermaNet® 2.0 LLINs distributed in a region of persistent malaria transmission in Guatemala was assessed by measuring their overall and functional survivorship, physical integrity, insecticide content and bio-efficacy.

The proportion of LLINs present in the household and in ‘serviceable’ condition was 69% after 32 months of routine use, with an extrapolated median survival time of 3.7 years. The median survival time reported in this study is higher than similar studies conducted in Africa that have also evaluated PermaNet® 2.0 LLINs. In Tanzania the estimated survival was 2.5 years, in Zanzibar it was 3.1–3.3 years and in Zambia it was 2.5-3 years [19, 21, 22]. In this study, the physical integrity of the LLINs—a key component for the functional survivorship—was high in each of the survey time-points (range: 84–94%), whereas other studies have reported lower values (e.g. 68% at 36 months) [22]. These differences are further highlighted by the median total hole area, which in this study was only 34 cm^2^ at 32 months, whereas other studies evaluating PemaNet® 2.0 LLINs reported values of 390 cm^2^ and 106 cm^2^ [15, 16]. In general, the size of the holes found on the LLINs in Guatemala was smaller, resulting in a greater number of LLINs categorized as being in ‘serviceable’ condition and a higher functional survivorship. The low proportion of new holes caused by thermal damage or animals as compared to other studies may also help explain these differences [23, 24]. It cannot be ruled out that the difference on hole size in this study compared to the ones in Africa might well reflect differences in what inhabitants accept, as in this population the main reason for attrition of nets between 24 and 32 months was discarded because of wear and tear.

Also, the overall survivorship of the LLINs was estimated to be 66% after 32 months. This was very similar to the functional survivorship, indicating that two thirds of the campaign LLINs were still present in the surveyed households nearly 3 years after their distribution. However, reasons explaining the overall and functional survivorship were different in the 18- and 24-month surveys. At these time points, the main reason reported for not having a net present in the household was because it had been ‘given away to others’, not because of wear and tear. These findings are similar to previous reports [25–27], highlighting the real-life challenges faced by national malaria programmes, which reach beyond the quality of the fabric of the nets. In a large study evaluating 14 household surveys from four African countries, 34% of the nets that were not present in the household had been given away, the majority to family members and within the first month after a distribution campaign [28].

In terms of usage and handling, between 71 and 79% of the users reported to have used the LLINs the night preceding the survey, with almost a third of the nets observed not being in use as they were not hanging in the sleeping place. Previous studies that have evaluated the usage of LLINs in Latin America have shown a variety of results around LLIN usage. In Colombia, the usage was 51.1% after 8 months, whereas in Venezuela, it was over 90% after 6 months, highlighting intra-regional differences [29, 30].

Decreases in insecticide content over time were detected, with a drop of 53% detected between unused nets (49.8 mg/m^2^) and nets at the 18-month survey point (23.2 mg/m^2^). These levels were lower than levels detected on PermaNet® 2.0 in Ethiopia, where the mean concentration of deltamethrin was 44.1 mg/m^2^ after 14–20 months of use [14]. Here, the proportion of nets with deltamethrin levels ≥ 10 mg/m^2^ after 32 months was 65%, a stark contrast to the 95% observed in the aforementioned study. The values obtained by XRF to estimate total insecticide content showed similar trends to the bioassays, where just 52% of the LLINs at 32 months met the WHO bioassay threshold of bio-efficacy. The frequent washing of the LLINs and the high proportion that were dried under direct sunlight are some of the factors that could have contributed to this decline [31, 32]. Over 50% of the nets reported to have been washed in the previous month of the survey. This might represent a frequent pattern of washing, resulting in the loss of the protective effectiveness of the nets against the mosquitoes [33].

As other studies have detected, it was found that a measurement of ≥ 25 mg/m^2^ by XRF was more likely to indicate a LLIN with a mortality at 24 h. of ≥ 80%, suggesting that this threshold is more closely associated with optimal bio-efficacy than 10 mg/m^2^ [14]. When the results of survivorship, physical integrity, usage and insecticide content were integrated, the estimated that the level of protection provided by the LLINs was sub-optimal. The overall proportion of LLINs providing adequate protection was only 38% after 24 months and dropped further to 21% at 32 months. National malaria data showed that the number of cases in Escuintla remained constant during the period of 2012–2015, suggesting that the LLINs did not provide sufficient protection.

Several lessons can be gathered from these findings. First, three key factors seems to contribute to the sub-optimal level of protection: the loss of LLINs still present in the household, the degree of usage of the LLINs, and the low percentage of LLINs with adequate insecticide content. Given that these factors are potentially compromising the protective effectiveness of the LLINs more than the durability of the fabric, activities focusing on community engagement and the inclusion of local civil society around LLIN use could be particularly impactful. Second, these findings suggest that personal redistribution of nets is an important trend in this region, similar as in Africa. By giving nets away to friends and family, communities may be compensating for inadequate campaign planning. National malaria programmes may consider including local leadership and shift toward a more decentralized approach to improve the design of LLIN campaign distributions [34]. Third, it has been shown that in areas with limited resources to carry out the WHO cone bioassay, the measurement of insecticide content by bromine x-ray fluorescence might be an accurate alternative methodology to estimate the bio-efficacy of LLINs.

A major strength of this study is that survivorship, physical integrity, insecticide content and bio-efficacy were concurrently measured. There are few data on these indicators reported from Latin America, and this study provides one of the most comprehensive evaluations to date for the Americas. In addition, by following standard WHO guidelines, these results can be compared to outcomes arising from similar studies conducted elsewhere, while at the same time including variables that were customized to the local context.

Thi study also had several limitations. The first one refers to the selection of the study households. During the 18-month survey, it was found that the MoH list did not provide enough information to locate the households in which LLINs were delivered. This resulted in a change on the household selection for the 24 and 32-month surveys, using GIS technology that provided a more accurate identification of the households in each community. The randomization process also limited the bias associated with the selection of the surveyed households. However, this resulted in a high non-response rate, in which again it was needed to rely on the information provided by the MoH list. Secondly, the XRF methodology detects bromine (atoms) and not deltamethrin directly. Several assumptions were made with this analysis, one being that the intensity of the emission is proportional to the concentration of deltamethrin in the sample. The experience of the authors is that these assumptions are generally true, but to confirm it will needed to measure the deltamethrin content of LLINS by X-ray fluorescence (calibrated using handmade samples) followed by the measuring the deltamethrin concentration of the same specimens using HPLC. As a final limitation, the answers for the questions related to usage, care and handling of the LLINs are prone to recall and social desirability bias [35].

## Conclusions

The lifetime of PermaNet® 2.0 is expected to be approximately 3 years. In this study, after 2 years, over two thirds of the LLINs did not contain the optimal deltamethrin concentration of 25 mg/m^2^ and 32% of them did not meet the WHO criteria for bio-efficacy. At 32 months, only one in five of the LLINs distributed in the campaign provided adequate protection in terms of survivorship, physical integrity, bio-efficacy and usage. The widespread provision of LLINs is a cornerstone of global efforts for the control and potential elimination of malaria, which is now a near-term goal in several Latin American countries [1]. It is recommended that future evaluations of the durability of LLINs should always include measurements of survivorship, usage and handling of the LLIN, physical integrity, and insecticidal activity (insecticide content and/or bio-efficacy) in order to estimate the protective effect of the nets in a community. The engagement of the National Malaria Programme with the community is critical to the success of future LLIN distributions in Guatemala.

## Supplementary Information

**Additional file 1: Table S1.** Additional descriptive characteristics of the households that were enrolled in the surveys at the three time-points. **Table S2.** Reasons for LLIN loss.**Table S3. **Association of deltamethrin (mg/m2) content level measured by X-ray fluorescence (XRF) and age of the long-lasting insecticidal nets (LLINs) in predicting the percentage of mosquito mortality within 24 hrs.** Table S4.** Estimate of effective protection of long-lasting insecticidal nets (LLINs) in La Gomera, Escuintla. **Figure S1.** Mortality at 24 hours of *Anopheles albimanus* (Sanarate strain) mosquitoes exposed in cone bioassays to long-lasting insecticidal nets (LLINs) at the surveys time-points. **Figure S2.** Knockdown after a 60-min (KD60) exposure of *Anopheles albimanus* (Sanarate strain) mosquitoes exposed in cone bioassays to long-lasting insecticidal nets (LLINs) at the surveys time-points. **Figure S3.** Locally weighted regression (LOESS) analysis between results of the cone bioassays measuring percent mortality at 24 hours and concentration of deltamethrin (mg/m2) as measured by X-ray fluorescence (XRF), stratified by surveys time-points (18 months, 24 months an 32 months).

## Data Availability

The data that support the findings of this study are available on request from the senior author, NP. The data are not publicly available due to containing information that could compromise the privacy of participants.

## Abbreviations

GIS: Geographic information system
GPS: Global positioning system
IQR: Interquartile range
ITN: Insecticide-treated bed net
LLIN: Long-lasting insecticidal net
LOESS: Locally Weighted Scatterplot Smoothing
MoH: Ministry of Health
PDA: Personal digital assistant
THSA: Total hole surface area
tm: Median survival time
WHO: World Health Organization
XRF: x-ray fluorescence
