# Supplementary material for: Evaluation of the durability of long‐lasting insecticidal nets in Guatemala
Source: Malar J. 2021 May 14;20:219. doi: 10.1186/s12936-021-03722-1 (PMC8120849; doi:10.1186/s12936-021-03722-1)
Supplement: Supplementary file 1 — Additional file 1: Table S1. Additional descriptive characteristics of the households that were enrolled in the surveys at the three time-points. Table S2. Reasons for LLIN loss.Table S3. Association of deltamethrin (mg/m2) content level measured by X-ray fluorescence (XRF) and age of the long-lasting insecticidal nets (LLINs) in predicting the percentage of mosquito mortality within 24 hrs. Table S4. Estimate of effective protection of long-lasting insecticidal nets (LLINs) in La Gomera, Escuintla. Figure S1. Mortality at 24 hours of Anopheles albimanus (Sanarate strain) mosquitoes exposed in cone bioassays to long-lasting insecticidal nets (LLINs) at the surveys time-points. Figure S2. Knockdown after a 60-min (KD60) exposure of Anopheles albimanus (Sanarate strain) mosquitoes exposed in cone bioassays to long-lasting insecticidal nets (LLINs) at the surveys time-points. Figure S3. Locally weighted regression (LOESS) analysis between results of the cone bioassays measuring percent mortality at 24 hours and concentration of deltamethrin (mg/m2) as measured by X-ray fluorescence (XRF), stratified by surveys time-points (18 months, 24 months an 32 months). [file 12936_2021_3722_MOESM1_ESM.docx]

SUPPLEMENTARY MATERIAL

Table Legends

Table S1. Additional descriptive characteristics of the households that were enrolled in the surveys at the three time-points.

Table S2. Reasons for long-lasting insecticidal (LLIN) loss.

Table S3. Association of deltamethrin (mg/m^2^) content level measured by X-ray fluorescence (XRF) and age of the long-lasting insecticidal nets (LLINs) in predicting the percentage of mosquito mortality within 24 hrs.

Table S4. Estimate of effective protection of long-lasting insecticidal nets (LLINs) in La Gomera, Escuintla. Results are shown for a) the overall study population, b) Data excluding LLINs with incomplete hole section and c) Data excluding LLINs with incomplete hole section and with no X-ray fluorescence (XRF) analysis. Results in bold are the ones presented in Figure 6.

Table S1. Additional descriptive characteristics of the households that were enrolled in the surveys at the three time-points.

| Characteristic | 18 months | 24 months | 32 months |
| --- | --- | --- | --- |
|  | n=290 | n=349 | n=349 |
| Number of people slept in household last night |  |  |  |
| Adults > 15 years (median, IQR) | 2 [2,4] | 2 [2,4] | 3 [2,4] |
| 5-15 years (median, IQR) | 1 [0,2] | 1 [0,2] | 1 [0,2] |
| <5 years (median, IQR) | 0 [0,1] | 0 [0,1] | 0 [0,1] |
| Total (median, IQR) | 4 [3,6] | 4 [3,6] | 4 [3,5] |
| Number of sleeping places used last night in your household (median, IQR) | 3 [2,4] | 3 [2,4] | 3 [2,4] |
| According to census, number of LLINs given by MoH in 2012 (median, IQR) | 3 [2,4] | 3 [2,5] | 3 [2,4] |
| Number of any bed nets in household that can be used for sleeping (median, IQR) | 4 [2,5] | 4 [2,5] | 3 [2,5] |
| Number of LLINs in household that can be used for sleeping (median, IQR) | 3 [2,4] | 3 [2,4] | 2 [1,3] |

Abbreviations: LLIN= long-lasting insecticidal nets, IQR = Interquartile boundary values (p25, p75)

Table S2. Reasons for long-lasting insecticidal (LLIN) loss.

|  | 18 months | 24 months | 32 months |
| --- | --- | --- | --- |
| Characteristic | N=290 | N=349 | N=349 |
| LLINs still present (n, %) | 249 (86) | 266 (76) | 229 (66) |
| LLINs not present (n, %) | 41 (14) | 83 (24) | 120 (34) |
| Reason for loss (n, %) |  |  |  |
| Net was given away to others (n, %) | 23 (56) | 45 (54) | 39 (32) |
| Net was damaged and thrown away (n, %) | 6 (15) | 21 (25) | 52 (43) |
| Net is being used in another location (n, %) | 5 (12) | 9 (11) | 18 (15) |
| Net was stolen (n, %) | 2 (5) | 2 (2) | 3 (2) |
| Do not know (n, %) | 2 (5) | 2 (2) | 3 (2) |
| Net being used for another purpose (n, %) | 1 (2) | 2 (2) | 0 (0) |
| Allergies (n, %) | 1 (2) | 0 (0) | 3 (2) |
| Unclear reason (n, %) | 1 (2) | 2 (2) | 2 (2) |

Abbreviations: LLINs=long-lasting insecticidal nets

Table S3. Association of deltamethrin (mg/m^2^) content level measured by X-ray fluorescence (XRF) and age of the long-lasting insecticidal nets (LLINs) in predicting the percentage of mosquito mortality within 24 hrs.

| Exposure variable (1st segment) | Estimate (se) | p-value |
| --- | --- | --- |
| Deltamethrin (mg*/*m^2^) in first segment | 2.8 (0.3) | <0.001 |
| 18 months | 27.1 (5.6) | <0.001 |
| 24 months | -18.8 (4.3) | <0.001 |
| 32 months | -24.9 (4.3) | <0.001 |

Association of Deltamethrin (mg/m^2^) in the 2nd segment was 1.1 (se = 0.5, CI95: 0.2, 2.0)

Table S4. Estimate of effective protection of long-lasting insecticidal nets (LLINs) in La Gomera, Escuintla. Results are shown for a) the overall study population, b) Data excluding LLINs with incomplete hole section and c) Data excluding LLINs with incomplete hole section and with no X-ray fluorescence (XRF) analysis. Results in bold are the ones presented in Figure 6.

| Criterion | 18 months | 24 months | 32 months |
| --- | --- | --- | --- |
| Overall data |  |  |  |
| Evaluated± (n, %) | **290 (100)** | **349 (100)** | **349 (100)** |
| Still present (n, %) | **249 (86)** | **266 (76)** | **229 (66)** |
| Used at least once (n, %) | **231 (80)** | **257 (74)** | **217 (62)** |
| Used last night (n, %) | **171 (59)** | **203 (58)** | **154 (44)** |
| Data excluding LLINs with incomplete hole section |  |  |  |
| Evaluated ^π^ (n, %) | 281 (100) | 348 (100) | 346 (100) |
| Still present (n, %) | 240 (85) | 266 (76) | 226 (65) |
| Used at least once (n, %) | 222 (79) | 256 (74) | 214 (62) |
| Used last night (n, %) | 164 (58) | 203 (58) | 152 (44) |
| Serviceable condition (based on total hole area) (n, %) | **151 (54)** | **174 (50)** | **126 (36)** |
| Data excluding LLINs with incomplete hole section and with no XRF analysis |  |  |  |
| Evaluated ^π π^ (n, %) | 91 (100) | 346 (100) | 335 (100) |
| Still present (n, %) | NE | 263 (76) | 215 (64) |
| Used at least once (n, %) | NE | 254 (73) | 204 (61) |
| Used last night (n, %) | NE | 201 (58) | 145 (43) |
| Serviceable condition (based on total hole area) (n, %) | NE | 172 (50) | 121 (36) |
| Over 10 mg/m^2^ (based on XRF analysis) (n, %) | NE | **133 (38)** | **72 (21)** |

Abbreviations: LLINs=long-lasting insecticidal nets, XRF = X-ray fluorescence

± This ‘n’ serves as the denominator for the overall data analysis.

π This ‘n’ serves as the denominator for the data excluding LLINs with incomplete hole section

π π This ‘n’ serves as the denominator for the data excluding LLINs with incomplete hole section and with no XRF measurement.

NE =Not estimated because only a small proportion of LLINs were analyzed by XRF analysis, so this measurement is likely to be biased.

SUPPLEMENTARY MATERIAL

Figure Legends

Figure S1. Mortality at 24 hours of *An. albimanus* (Sanarate strain) mosquitoes exposed in cone bioassays to long-lasting insecticidal nets (LLINs) at the surveys time-points. The time-point of 0 represents values on unused LLINs that were from the same batch as those distributed. Each box represents the interquartile distance, center line in each box indicates the median. The black dots indicate each of the values of a LLIN. The dashed horizontal line represents the 80% mortality threshold.

Figure S2. Knockdown after a 60-min (KD60) exposure of *An. albimanus* (Sanarate strain) mosquitoes exposed in cone bioassays to long-lasting insecticidal nets (LLINs) at the surveys time-points. The time-point of 0 represents values on unused LLINs that were from the same batch as those distributed. Each box represents the interquartile distance, center line in each box indicates the median. The black dots indicate each of the values of a LLIN. The shaded horizontal line represents the 95% KD60 threshold.

Figure S3. Locally weighted regression (LOESS) analysis between results of the cone bioassays measuring percent mortality at 24 hours and concentration of deltamethrin (mg/m^2^) as measured by X-ray fluorescence (XRF), stratified by surveys time-points (18 months, 24 months an 32 months). Each dot represents one long-lasting insecticidal net (LLIN) with their corresponding cone bioassays and XRF measurements. The gray area represents 95% confidence interval. The solid vertical line represents the threshold of 10 mg/m^2^ and the dashed vertical line represents the threshold of 25 mg/m^2^. The black horizontal line represents the 80% mortality threshold.

Figure S1. Mortality at 24 hours of *An. albimanus* (Sanarate strain) mosquitoes exposed in cone bioassays to long-lasting insecticidal nets (LLINs) at the surveys time-points. The time-point of 0 represents values on unused LLINs that were from the same batch as those distributed. Each box represents the interquartile distance, center line in each box indicates the median. The black dots indicate each of the values of a LLIN. The dashed horizontal line represents the 80% mortality threshold.


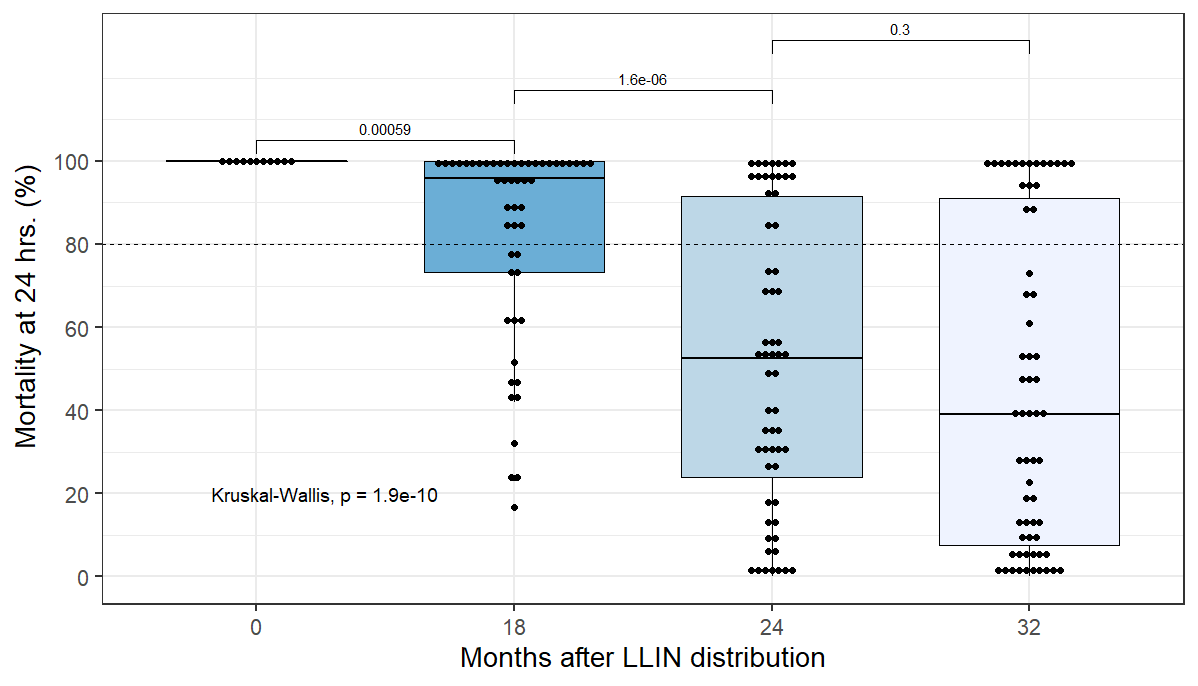


Figure S2. Knockdown after a 60-min (KD60) exposure of *An. albimanus* (Sanarate strain) mosquitoes exposed in cone bioassays to long-lasting insecticidal nets (LLINs) at the surveys time-points. The time-point of 0 represents values on unused LLINs that were from the same batch as those distributed. Each box represents the interquartile distance, center line in each box indicates the median. The black dots indicate each of the values of a LLIN. The shaded horizontal line represents the 95% KD60 threshold.


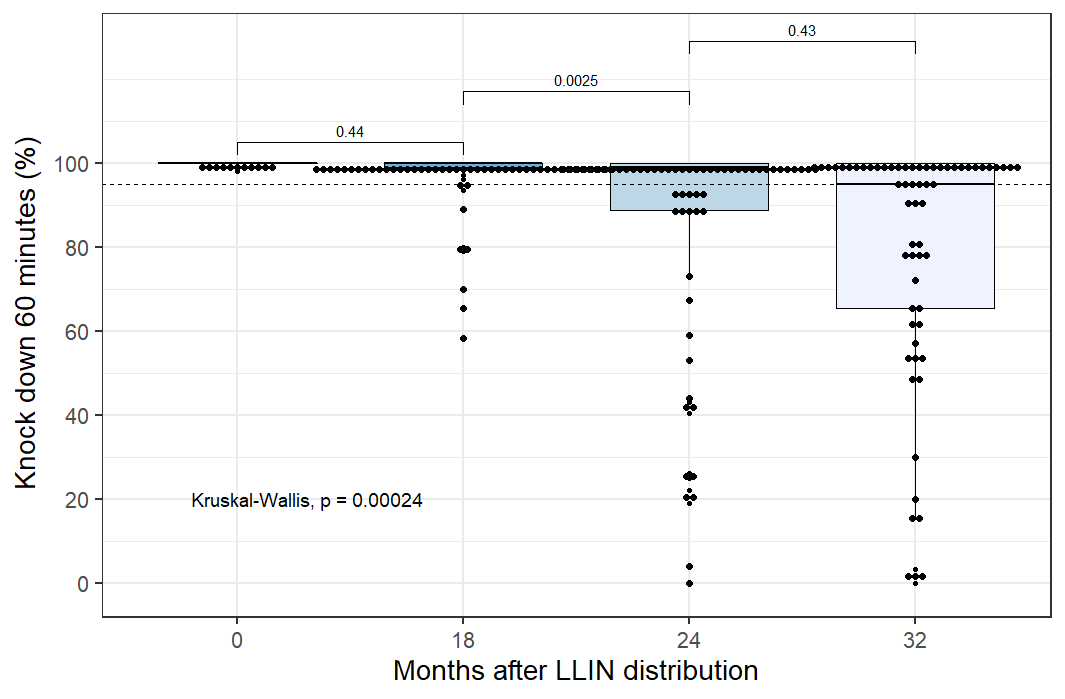


Figure S3. Locally weighted regression (LOESS) analysis between results of the cone bioassays measuring percent mortality at 24 hours and concentration of deltamethrin (mg/m^2^) as measured by X-ray fluorescence (XRF), stratified by surveys time-points (18 months, 24 months an 32 months). Each dot represents one long-lasting insecticidal net (LLIN) with their corresponding cone bioassays and XRF measurements. The gray area represents 95% confidence interval. The solid vertical line represents the threshold of 10 mg/m^2^ and the dashed vertical line represents the threshold of 25 mg/m^2^. The black horizontal line represents the 80% mortality threshold.


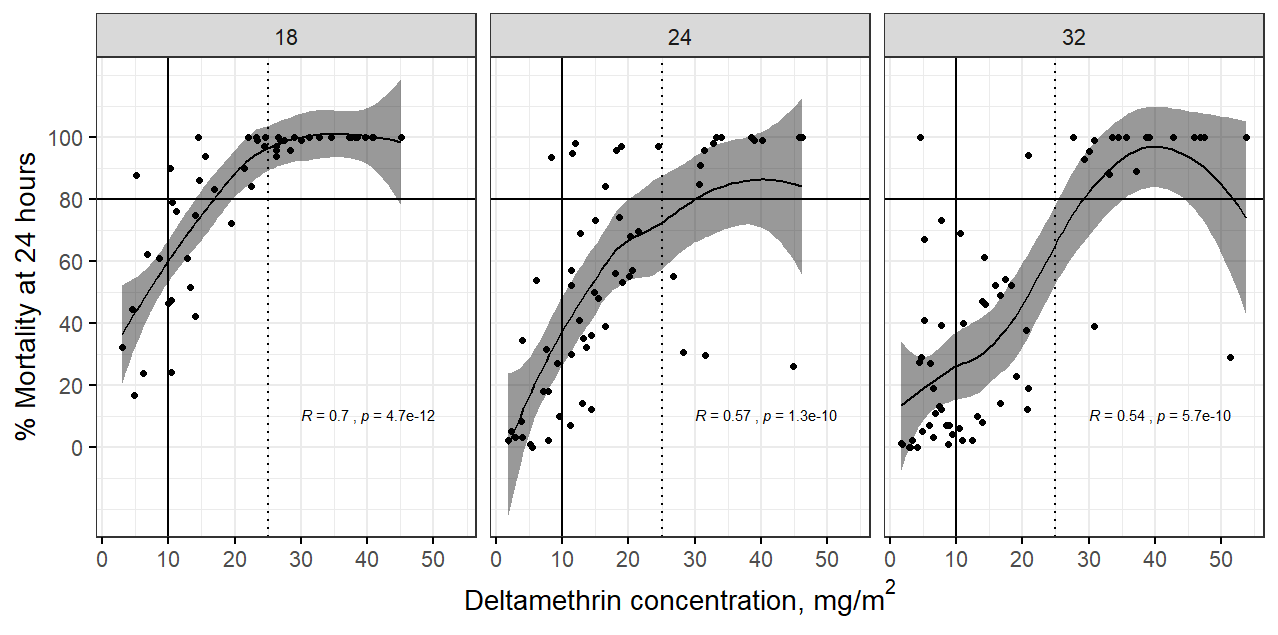


APPENDIX FILE

**Methodology to assess if insecticide content measurement obtained by XRF could serve as a predictor to estimate the mortality at 24 hours.**

- We developed a predictive model using piecewise (segmented) regression to accommodate the change in the magnitude and direction of the linear relationship between the outcome and exposure variables observed in our exploratory analysis.

We use selected responses from the surveys as exposure variables:

- Months after distribution of LLIN
- Highest level of education of the head of the household
- Net still present in the household
- Net used the night before of the survey to sleep under
- Frequency of the net used in the week before of the survey to sleep under
- Adult (>15 years) slept under the net the night before of the survey
- Children (5-15 years) slept under the net the night before of the survey
- Children (<15 years) slept under the net the night before of the survey
- Net has been washed
- Last time the net was washed
- Type of soap used to wash the net
- Net was soaked when washing
- Length of time a net was soaked
- Net was scrubbed when washing
- Location were net was dried
- Presence of open flame were the net was located
- Total area of holes of LLIN
- Condition of LLIN based on holes area (Good, damaged or too torn)
- Deltamethin content (mg/m^2^) measured by XRF
- Outcome variable: % Mortality at 24 hours
- The model was first trained using 80% of the data and validated in the remaining 20%. We used random sampling to separate the data as training and test data. This process was repeated 1000 times. To assess the accuracy of the fitted model, a confusion matrix was built to calculate the proportion of correct classification when the model was applied to the test data
